# Optimization of preparation parameters of ceramic pot water filters for potential application of microbial and fluoride removal from groundwater

**DOI:** 10.1016/j.heliyon.2023.e13261

**Published:** 2023-01-26

**Authors:** Alemayehu Solomon, Dinsefa Mensur Andoshe, Adane Muche Abebe, Tatek Temesgen Terfasa, T. Ganesh, Fekadu Gashaw Hone, Newayemedhin A. Tegegne, Belay Brehane Tesfamariam

**Affiliations:** a Materials Science and Engineering Department, Adama Science and Technology University, Adama, 1886, Ethiopia; b Chemical Engineering Department, Adama Science and Technology University, Adama, 1886, Ethiopia; c Physics Department, Addis Ababa University, Addis Ababa, 1176, Ethiopia

**Keywords:** Ceramic filter, Defluoridation, Microbial removal, Water filter, Clay

## Abstract

The need to make clean water accessible and affordable for low-income countries is crucial. This study examines the suitability of various clays for Ceramic Pot Water Filters production and groundwater treatment for effective microbe and fluoride removal. For this study, three clays were collected from different geographical locations in Ethiopia,i.e., Hosaenna Clay, Babawuha Clay, and Leku Clay. Organic additives such as sawdust and eragrostis tef husks were used to increase the porosity of the Ceramic Pot Water Filters. The Atterberg limit and particle size distribution tests revealed that BC and HC have moderate to high plasticity and mouldability, making them suitable for CPWF production. The clay chemical composition, phase analysis, and thermal properties were determined using XRF, XRD, and TGA/DTA. The turbidity, fluoride level, total dissolved solids, and pH of the groundwater decreases, from 13 to 0.45 NTU, from 3.4 to 0.053 mg/100 mL, from 1245 to 360 mg/l, and from 8.4 to 7.3, respectively; all of which are within the acceptable range of WHO drinking water standards. Microbial removal tests show that the CPWFs removed 99.3%–100% of total coliform bacteria and 98.48%–100% of fecal coliform bacteria from groundwater. Therefore, this work paves the way to fabricate a clay-based ceramic water filter for low-income countries to provide affordable household groundwater treatment technology for microbial and excess fluoride removal.

## 1 Introduction

Water is a basic need to sustain life. A safe, reliable, affordable, and easily accessible water supply is essential for good health [1]. Currently, about 1.8 billion people do not have access to clean water, majorities who live in rural areas of developing countries [2,3]. Based on the World Health Organization (WHO) report, due to the lack of safe drinking water, about 3.4 million people, including children, die annually from waterborne diseases [2,4]. Out of this number, 2.2 million people die from diarrhea and cholera [5]. Therefore, there is a need to make accessible and affordable safe water for low-income communities; that are vulnerable to waterborne diseases. To alleviate this challenge, household water treatments and safe-storage mechanisms are among the viable solutions.

Fluoride is a naturally occurring element and nearly insoluble in water. It can be found in minerals, geochemical deposits, and natural water systems and enters food chains by drinking groundwater or eating plants and cereals [4,6]. It occurs naturally as MgF_2_, CaF_2_, Na_3_AlF_6_ and 3Ca_3_(PO_4_)_2_Ca (F, Cl_2_) [6]. According to the WHO, the tolerable limit of fluoride content in drinking water is 1.5 mg/l [7]. Fluoride concentration ranges from 1.5 to 4 mg/l resulting in dental fluorosis, whereas prolonged exposure to 4–10 mg/l causes skeletal fluorosis. Methods and materials used for the removal of fluoride from drinking water are mainly membranes that can filter fluoride and adsorbents such as alumina-based, clays and soils, carbon, zeolites, and layered double hydroxides [4]. Removal of fluoride using a membrane is more expensive than adsorbents. Therefore, fluoride removals using adsorbent are effective and widely used method due to low maintenance cost and appreciable fluoride removal.

Clay minerals such as kaolinite and bentonite adsorbents remove about 50% of fluoride from water. Moreover, the coatings on these minerals have shown better potency in removal at a pH of 6; for example, kaolinite coated with aluminum oxide, iron oxide, and without coating showed 72.3%, 61.3%, and 45.1% fluoride adsorption efficiency, respectively. Moreover, bentonite coated with aluminum oxide and iron oxide showed fluoride adsorption efficiency of 95.3% and 80.4%, respectively [[8], [9], [10]]. Kaolin has high chemical stability, better cation exchange capacity, and a low expansion coefficient [11]. The better cation exchange capacity of kaolinite shows good performance in lead ions removal from an aqueous solution [12].

Ceramic pot water filter (CPWFs) seems viable solutions for efficient removal of microbes and fluorine from unsafe water for low income community [[13], [14], [15]]. Usually, Clay-based ceramic Water Filters have prepared by mixing clay and water. Organic materials such as sawdust (woodchips), rice husk, coffee husk, and flour are added to the mix to enhance the porosity of the filter for a better water discharge rate [16]. The mixed clay with water and organic materials fired at an elevated temperature, and it became mechanically strong, chemically stable/inert, and possesses porosity [1,[17], [18], [19]]. The water discharge rate, defluoridation, and microbe removal efficiency of CPWF depend on the composition of the clay, firing temperature, particle size, ramming (manual or hand consolidation/forming) pressure, additives, and the reaction occurring on firing [20].

However, though there are few works of literature reported on the potential application of CPWF for water purification, the authors are not aware of prior comprehensive studies to optimize the organic additives, clay properties, clay-to-organic additive ratio, and firing temperature that determine the water discharge rate, pathogen, and fluoride removal performance of the CPWF.

Herein, the suitability of Leku, Hosana, and Babawuha clay (Southern Ethiopian) to prepare CPWF is studied. The fluoride and pathogenic microbes removal efficiency of the CPWF from contaminated groundwater sources are measured. Using different ceramic processing parameters such as compositions of clay and combustible organic additive materials, sawdust, and eragrostis tef husk are used to optimize the water absorption and apparent porosity of the resulting CPWF to improve its water discharge rate.

## 2 Materials and methods

### 2.1 Analysis of Ethiopian clay

Among different types of clay soils available in Ethiopia, three clay soils from three different locations are tested. The clay soils used in this study are; Babowuha Clay (BC) from Guji zone (6*°* 05′ 20″ N and 38*°* 46′ 30” E) in the Oromia region of Ethiopia, Hosanna Clay (HC) from Hadiya zone (7*°* 32′ 60″ N and 37*°* 52′ 60″ E) and Leku Clay (LC) from Shebe-dino zone (6*°* 52′ 23.12″ N and 38*°* 26’ 39.30″ E) of Sidama in Southern part of Ethiopia. Moreover, to enhance the porosity of the resulting CPWF, organic combustible material additives are used from eucalyptus hardwood sawdust from private timber manufactures in Adama town and brown eragrostis tef husk from Awash Melkassa Agricultural research institute, Ethiopia.

Particle size distribution (PSD) of the clays collected from the three different areas mentioned above, were measured by using both sieve and hydrometer analysis, according to American Standard Test for Materials, ASTM D 422 standard testing method (ASTM D422, 2007). The sieves used in this study for particle size distribution analysis is seven stacked sieves from 4 to 200 mesh size fitted with automatic mechanical sieve shaker, Casagrande apparatus and ASTM 152H type hydrometer. The Atterberg limits such as shrinkage limit, Plastic Limit (PL), and Liquid Limit (LL) are calculated according to the procedure of the ASTM-D 4318–10. The range of moisture content in which the soil remains plastic is determined by Plasticity Index (PI) as shown in equation (1).

Eq.1 PI=LL−PL

Crystallinity and phase purity of the clay was measured using powder XRD; model Dangil Shimadzu XRD-7000S. The X-Ray Fluorescence spectroscopy, model XRF-Thermo Fisher ARL9400 XP+ was used for chemical composition analysis of the clays. The Differential Thermogravimetric (DTG) analysis, using Dangil Shimadzu DTG-60 was used to examine thermal properties of the clays and organic material additives.

### 2.2 Preparation of CPWF

The CPWFs were prepared by using an aluminum mold with a base diameter (Db) = 3.4 cm, top diameter (DT) = 5.7 cm, height (h) = 5.4 cm, and thickness of the lip (L_lip_) = 1.5 cm and angle of filter wall inclination (θ) = 80°; designed and manufactured by Zenebe metal work PLC Adama, Ethiopia. An oven and muffle furnace was used to fire the prepared sample CPWF.

The samples were prepared from the clay powder sieved by using sieve diameters of 250 μm and 595 μm, and the combustible materials were sun-dried for two days and later crushed, ground, and sieved by diameter of 595 μm and 1190 μm sieve.

The clays and combustibles were mixed according to the optimal mixing range evaluated from the optimum processing parameter. The homogenous mixture was pressed into a pot shape using a manually driven hydraulic filter press with a purpose-built aluminum mold applying 5kgft/cm2 bumping pressure per each filter piece sample with a 2-min holding time.

Finally, physical shape, quality, and surface finishing are checked using a scrubbing tool. After shaping, the filters were open-air-dried for 24 h and oven-dried for two days to remove moisture. Finally, the filters were fired at 900 °C in a ceramic firing muffle furnace with a firing rate of 45–100 °C/h followed by furnace cooling to room temperature.

### 2.3 Filter performance evaluation

Three major water filter performance tests namely filtration rate, physiochemical test (conductivity, pH, water hardness, turbidity, Total Dissolved Solids (TDS) and fluoride removal test) and microbial removal (Total Coliform (TC) and Fecal Coliform (FC) tests for the prepared CPWFs was performed.

A locally available raw groundwater sample was collected from Gedemssa and Kelbo village, which is found nearby Wonji town, Ethiopia. The raw groundwater was collected by using a pre-sterilized (120 °C) 1-L plastic bottle and sealed. The groundwater sample was packed in the icebox containing brushed ice 31 at 4 °C and transported to Oromia Self-Help Organization (OSHO) water quality analysis laboratory.

#### 2.3.1 Filtration rate testing

To obtain a sufficient water discharge rate from the CPWF, optimizing the process parameters while manufacturing the ceramic filter is necessary. Therefore, the parameters such as the mixing proportion, amount of water, types of raw materials, and firing temperature are optimized by shrinkage, porosity, and water of plasticity data. The water of plasticity, shrinkage, and porosity of the CPWFs was analyzed according to the Pottery For Peace (PFP) standards [21,22].

The amount of water required in the clay & combustible mixture to become workable of the desired consistency is called water of plasticity (*w*) and is calculated by using Eq. (2).

Eq.(2) $$w=\frac{w_{w}}{w_{dp}}\times 100\%$$

where *w* is water of plasticity, *w*_w_ is weight of wetting water and *w*_dp_ is weight of dry powder.

Shrinkage such as dry/linear shrinkage (*S*_L_), firing shrinkage (*S*_F_) and total shrinkage (*S*_T_) of the samples were determined according to ASTM C 326–03 and Eqs. (3)–5).

Eq (3) $$S_{L}=\frac{L_{p}-L_{d}}{L_{p}}\times 100\%$$

Eq.(4) $$S_{F}=\frac{L_{p}-L_{f}}{L_{d}}\times 100\%$$

Eq.(5) $$S_{T}=\frac{L_{p}-L_{f}}{L_{p}}\times 100\%$$

where, *L*p is plastic bar length, *L*_d_ is dry bar length, *L*_f_ is fired bar length,

The degree of water absorption of the fired clay or percentage of porosity (*P* %) were determined using the Archimedes liquid displacement method based on ASTM C373-88. Then the porosity of the sample was calculated by using Eq. (6):

Eq.(6) $$P\%=\frac{{BW}_{sat}-{PW}_{d}}{{PW}_{d}}\times 100\%$$

where BW_sat_ is saturated bar weight and PW_d_ is dry powder weight.

#### 2.3.2 Physiochemical test

Physiochemical tests for the CPWFs were measured before and after the groundwater filtration. For this purpose, Ω metrohm 704 pH meters and portable digital conductivity meter CC-401 were used to determine the pH and electrical conductivity, respectively. The turbidity and fluoride concentration test were measured using a portable digital Photometer 7100. Titration method used to measure the water hardness and total dissolved solids (TDS) before and after filtration.

#### 2.3.3 Microbial removal test

The microbiological removal efficiency was tested using membrane filtration method using Lauryl Sulphate Tryptose (MLSB) broth (Wagtech, England) culture medium, absorbent pad and 0.45 μm portable Millipore membrane filter, incubator (Millipore, XX631K230), and analytical grade reagents. The counting was done by magnifying glass. The result of triplicate test is reported for this research.

## 3 Result and discussion

### 3.1 Physical properties of Babawuha, Hossana and Leku Clay

**Fig. 1:**
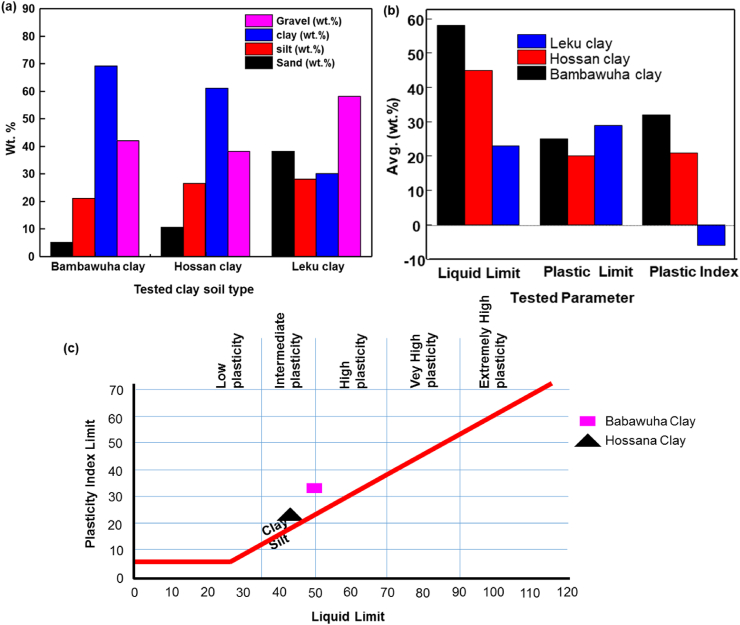
(a) Average weight percentage of fine clay particles and gravel, (b) The liquid limit, plastic limit and plastic index and (c) Bain and Highley plasticity chart for determination of plastic behavior for BC, HC and LC.

The sieve analysis result for the particle size distribution of the three clay types is presented in Table 1 and Fig. 1a.

**Table 1:** Sieve analysis of clays used in this study.

Soil Type	Diameter of the fine clay (μm)	Diameter of the gravel (μm)
BC	≤ 2	25–30
HC	≤ 2	≤ 50
LC	0.5	30–34

BC has the highest percentage of fine particles, with a diameter of about 2 μm than HC and LC clay. However, LC has the highest gravel percentages with a diameter of 34 μm compared to BC and HC, as shown in Fig. 1a and Table 1. Based on the Unified Soil Classification System and ASTM Standard D2487 - 10 analysis [23] (ASTM D2488-09a, 2009), The LL of BC greater than HC and LC with an average of 58%, 45%, and 23%, respectively. Moreover, the PL and PI of the clays are shown in Fig. 1 (b).

Plasticity is an important parameter during the forming stage of ceramic fabrication. It also affects the mechanical properties and creates product failure due to poor heterogeneity [[23], [24], [25], [26]]. The workability graph of plasticity index vs. liquid limit was determined according to Bain and Highley, as shown in Fig. 1c.

Therefore, based on the Bain and Highly plasticity chart, the BC soil has high plasticity because its LL and PI are 58% & 32%, respectively. HC soil shows an intermediate plasticity nature with 45% LL and 21% PI. The low liquid limits and below zero plasticity index of LC soil shows its non-plastic nature due to having quartz with coarse sand at a relatively larger grain size. Hence, the LC cannot describe using the plasticity chart shown in Fig. 1c [27]. The plasticity nature of BC & HC gives sufﬁcient workability to maintain body strength during the plastic forming stage without failure; therefore, BC & HC are reasonable to use for CPWFs production.

### 3.2 Optimum parameters for CWPFs preparation

The water plasticity and shrinkage of the prepared pellets having different weight percent composition of clay and organic additives were analyzed to find the optimum composition for the production of CWPF using equations (2)–(5).

#### 3.2.1 Water of plasticity

**Fig. 2:**
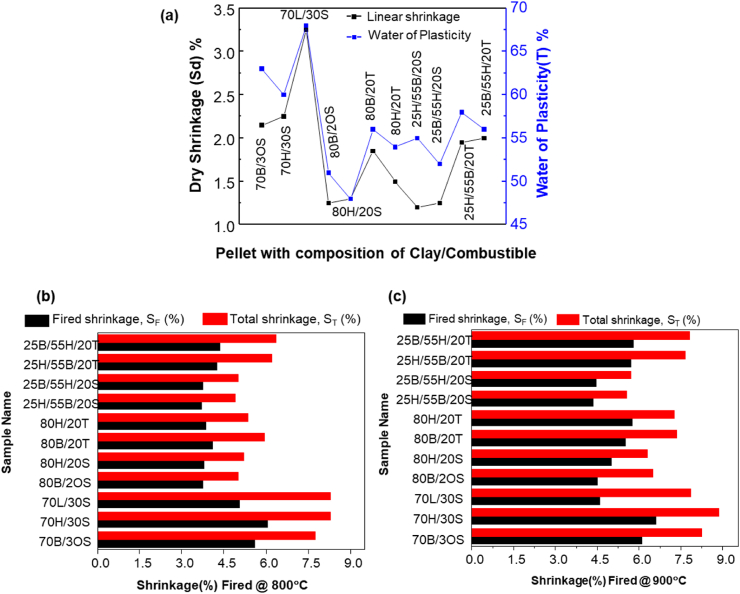
Shrinkage and water of plasticity of Pellets for different composition (a) Dry shrinkage and water of plasticity, (b) Fired and total shrinkage test bar samples fired at 800 °C, (c) Fired and total shrinkage test bar samples fired at 900 °C.

Around eleven test pellets made by different clay compositions and organic additives have been evaluated for their water of plasticity, as shown in Fig. 2a. The prepared test pellets are named considering the clay and filler ratio; for example, the “XH/YS” pellet represents the “X” weight percent of H (H represents hosanna clay) and the “Y” weight percent of S (S represents sawdust) in the composition. Pellets prepared with the BC composition have higher water of plasticity than the same composition pellets made from HC, and pellets prepared from the LC composition possess higher water of plasticity than the pellets prepared with an identical amount of HC and BC composition.

Eragrostis tef husk (T) -based test pellets have shown higher water plasticity than sawdust-based test pellets of the same formulation. The average water plasticity for the test pellets ranged from 48% to 68%, as shown in Fig. 2a. The high water plasticity is mainly due to the higher proportion of the combustible organic materials, forced to use high mixing water to maintain test pellet workability. Test pellets with a 30% composition of combustibles materials and LC showed low workability and lower mechanical integrity and failed during the experimentation.

However, the test pellets with a formulation of 20% of combustible materials gave the best combination of properties such as optimum plasticity nature, mechanical strength, and total shrinkage. Therefore, to get better workability and maintain the green body consistency, the filter composition with 80 wt percentages of BC & HC and 20 wt percentages of combustible materials was selected for CPWFs production.

#### 3.2.2 Shrinkage test results

Eleven test pellets were fired at 800 °C and 900 °C and analyzed for their percentage of shrinkage. The average linear drying shrinkage (S_L_) of the test pellets varied from 1.25% to 3.25%, the highest S_L_ obtained for the LC-based sample.

The average fired shrinkage varied from 3.35% to 6.6%, and the shrinkage percentage increased when the firing temperature increased, as shown in Fig. 2 (b)–(c).

Similarly, the total shrinkage (S_T_) for test pellets samples varies from 4.90% to 8.85%. The result shows a direct relationship between shrinkage and the percentage of combustible material added to the test pellet. The firing and total shrinkage of the sample pellet generally increased as the weight percent of filler materials in the sample pellet increased.

The percentage of total shrinkage generally decreases with an increased proportion of BC and HC in the test pellets formulation. Most test pellets prepared from LC and having higher combustible filler showed lower workability and mechanical integrity, and some of these test pellets failed during the experimentation process.

As an indicator of the firing of clay samples, the recommended total shrinkage value of less than 15% is more desirable for the clay to be less susceptible to volume change and cracking after and during the firing stage [28]. Similarly, the recommended linear shrinkage range for clays is less than 10% for the fabrication of ceramic water filters [29]. Based on these criteria, the tested pellets composed of higher weight percentages of BC and HC, which falls within the recommended ranges value, were selected for ceramic water filter preparation.

### 3.3 Crystallographic phase, chemical composition & thermal properties of the clay and organic additives

#### 3.3.1 X-ray diffraction analysis

**Fig. 3:**
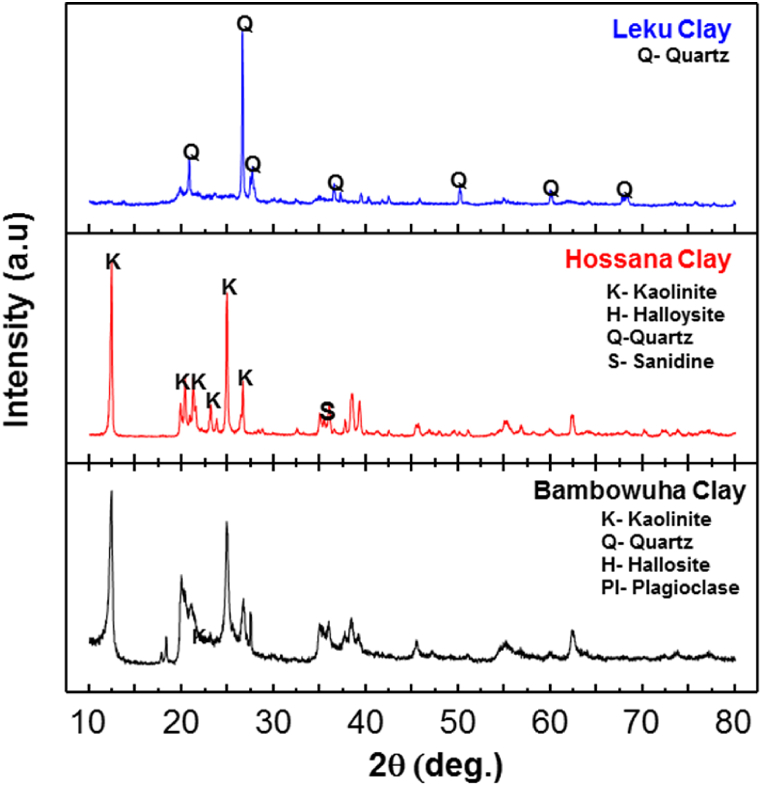
X-ray diffraction pattern of LC represented by blue color (top), HC represented by red color (middle) and BC represented by black color (bottom). (For interpretation of the references to color in this figure legend, the reader is referred to the Web version of this article.)

The X-ray diffraction of the BC and HC indicated that the clays are predominantly composed of kaolinite with the chemical formula Al_2_Si_2_O_5_(OH)_4_ (ICDD 00-058-2030 and 00-058-2030, respectively). Peaks for minerals including quartz SiO_2_ (ICDD 00-046-1045), microcline (k-feldspar) KAlSi_3_O_8_ (ICDD 01-084-0709), and halloysite KAl_2_(AlSi_3_)O_10_(OH)_2_ (ICDD 00-058-2006) observed, as shown in Fig. 3. The obtained XRD data is consistent with the previous literature [30,31] except for the slight mineralogical difference between BC and HC obtained. HC has sanidine as a secondary phase. The LC is pure quartz-SiO_2_ (ICDD 00-046-1045) (100 wt %) as confirmed by XRD, the physical characteristics of particle distribution analysis, and the Atterberg limit tests. Leku soils have sandy average particle size distribution and non-plastic nature. Therefore, the LC has minimum mouldability nature, and it cannot maintain its body strength during the plastic forming stage, which makes it fragile during the forming stage of filter production. Hence, LC is not suitable for CPWF fabrication.

#### 3.3.2 XRF chemical composition analysis

The XRF analysis was done for both BC and HC but not for LC. Because LC has already confirmed that LC has pure quartz but not clay. Table 2 shows the percentage of oxides composition in the BC and HC as obtained from The XRF. The potassium oxides, sodium oxides, and calcium oxides detection shows the feldspar (microcline & plagioclase) presence in the clay. Similarly, magnesium detection in the clay is probably an indication of the availability of muscovite/illite (mica minerals) in clay.

**Table 2:** XRF data for BC and HC.

Clay Raw Materials	Oxides wt.%									
	SiO_2_	Al_2_O_3_	Fe_2_O_3_	CaO	MgO	Na_2_O	K_2_O	P_2_O_5_	TiO_2_	LOI
BC	46.86	36.74	0.86	0.02	0.08	0.01	1.34	0.04	0.01	13.85
HC	46.70	37.50	0.14	0.09	0.01	0.01	0.01	0.01	0.57	14.6

Where LOI is the Loss on Ignition.

The XRF result showed that BC and HC have high Loss On Ignition (LOI) of 13.85 and 14.6 wt %, respectively. This is due to the loss of structural hydroxide groups that occurred during the transformation of kaolinite clay to meta-kaolinite phase formation above the firing temperature of 500 °C [30]. The amount of quartz found in BC and HC is sufficient for the ceramic water filter preparation due to its high percentage of filler oxides (feldspar & halloysite in the clays and sanidine in HC). Moreover, from the PSD analysis and Atterberg limit results; BC and HC possess fine clay with moderate to high plasticity and mouldability, which makes them suitable for CPWF production. Therefore, in terms of chemical composition, the BC and HC meet the chemical composition requirement of clays for ceramic water filter fabrication.

#### 3.3.3 Differential and thermo-gravimetric analysis

**Fig. 4:**
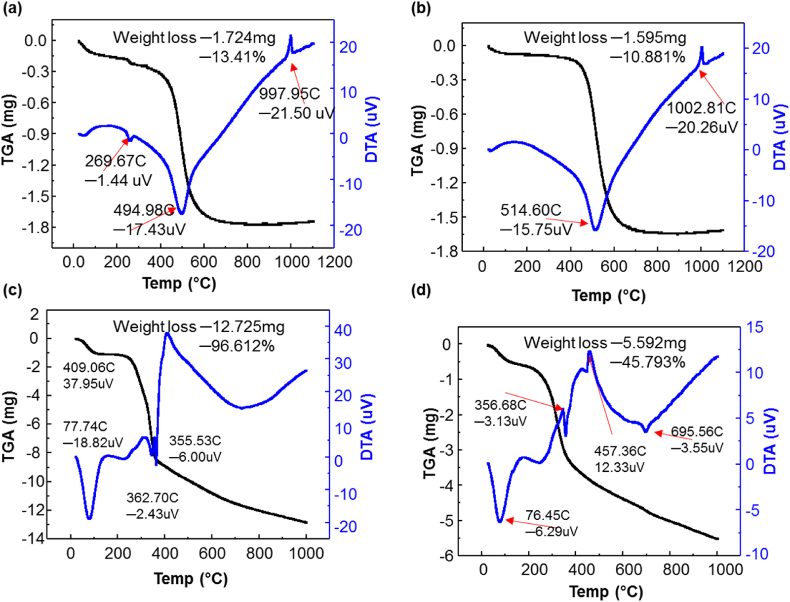
TGA/DTA curve for (a) BC, (b) HC, and (c) Eucalyptus hard wood saw dust, and (d) brown eragrostis tef husk. (For interpretation of the references to color in this figure legend, the reader is referred to the Web version of this article.)

For BC, HC, an eragrostis tef husk (T) & saw specks of dust TGA/DTA test were done and shown in Fig. 4. The DTA curve of BC/HC shows endothermic peaks at around 55 °C/60 °C, 269.67 °C, and 494.98C/514.60 °C, and Exothermic peaks were observed at 997.95 °C/1002.81 °C, respectively. The endothermic peaks arise probably due to the physically adsorbed or weakly-bound hydrogen of water and volatile organic compounds removed. The free gibbsite sheets and mineral dehydroxylation, Al_2_O_3_.2SiO_2_.2H_2_O, might be the cause of the endothermic peaks [[32], [33], [34]]. Similarly, the exothermic peaks at 997.96 °C and 1002.81 °C, observed for BC and HC, respectively, are due to the metakaolinite recrystallization/structural rearrangement in the spinel phase. The TGA/DTA measurements show BC and HC weight loss of 13.41% and 10.88%, respectively, as shown in Figures (a) & (b).

The highest weight loss happened between 250 °C and 600 °C for both combustible materials (sawdust and eragrostis tef husk), as shown in Fig. 4 (c) & (d). Therefore, the holding firing temperature is taken beyond 400 °C and chosen by default to be 900 °C for 2 h during the firing stage of the CPWF fabrication process. At the selected holding firing temperature, the combustible materials are completely burnout by leaving enough pores throughout the filter body. However, for the CPWF, which has Eragrostis tef husk as an organic additive, it was difficult to see the complete removal, as shown in Fig. 4d. The mass loss vs. temperature data shows that brown Eragrostis tef husk lost only 45% of the mass after being fired at 900 °C, the remaining 55 wt % might be silica [35], and it stayed in the CPWF.

### 3.4 Filter performance

BC and HC have selected based on the physical test, water plasticity, and shrinkage test results for ceramic water filter preparation. Eight CPWF with different clay and combustible materials compositions, 80H/20S1,80H/20T, 25B/55H/20S, 25B/55H/20T, 80B/20S, 80B/20T, 25H/55B/20S and 25H/55B/20T has prepared. The CPWF filtration rate, chemicals, and microbial removal efficiency are measured.

#### 3.4.1 Filtration rate

**Fig. 5:**
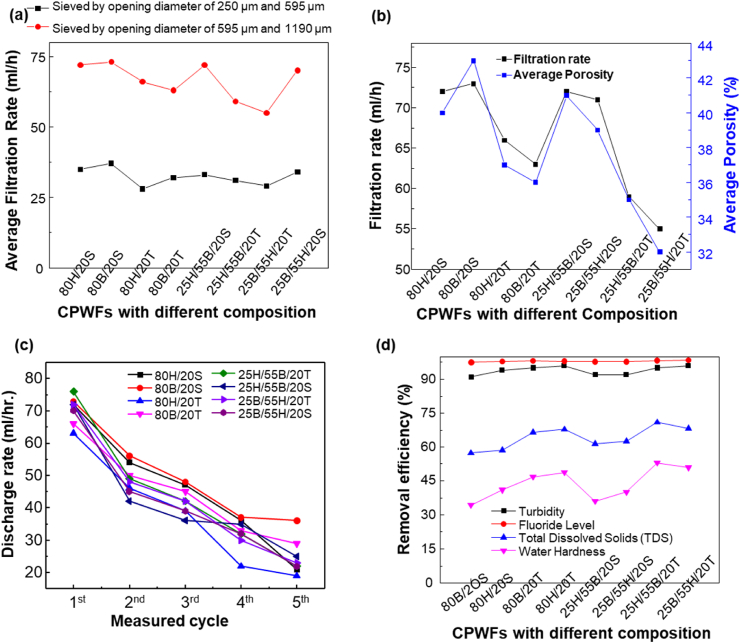
Filter rate and physiochemical removal performance test of CPWF (a) Effect of particle size on discharge rate, (b) Porosity vs. discharge rate sieved by 595 μm and 1190 μm opening dimeter, (c) Discharge rate, (d) physiochemical removal efficiency.

Fig. 5 depicts the effect of particle size, composition, and type of clay and combustible matter on the discharge rate of CPWFs. The clay and combustibles additive's particle size affects the discharge rate of CPWFs. The 5-h mean discharge rate of the CPWF made from clay and combustibles materials sieved by a mesh having an opening diameter of 595 μm and 1190 μm was higher for all compositions than for mesh having an opening diameter of 250 μm and 595 μm, as shown in Fig. 5a.

The sawdust-containing CPWF has an average higher discharge rate than Eragrostis tef husk because the sawdust is completely burned out from the CPWF during firing, resulting in relatively greater porosity for the filter, as shown in Fig. 4 (c) (d) and Fig. 5b.

The obtained discharge rate measured for 3 h in five consecutive cycles shows that the water discharge rate decreases when the operation time is longer, as shown in Fig. 5c.

All the prepared CPWF shows a remarkable discharge rate at the first operating cycle hours. However, the water discharge rate continuously decreases. Reasons for the decrease in the water discharge rate as the operation time increases could be due to pores clogging for longer flow durations and water level decrease, and the pressure in the filter [[36], [37], [38]].

The average percentage porosity for the CPWF ranges from 32% to 43%. The highest porosity and water discharge rate belongs to the CPWF prepared by incorporating sawdust due to the complete burned out from the sample confirmed by the result of TGA/DTA.

The discharge rate of the CPWF made from BC is higher than that of the HC. The highest average discharge rate in the five cycle measurements of each CPWF for 25 h was 39 mL/h for the 80BCE/20S composition, and the lowest was 26 mL/h for the 80HC/20T filter composition.

#### 3.4.2 Chemical contaminants removal test

The average chemical contaminants removal of the CPWFs, i.e., turbidity, fluoride level, total dissolved solids (TDS), water hardiness and electrical conductivity, and other parameters before and after filtration, are measured and summarized with the maximum permissible level of WHO standards in Table 3.

**Table 3:** The average chemical contaminant removal results of CPWFs fired at 900 °C.

Tested physiochemical parameters	Before filtration	After filtration								Maximum permissible level for drinking water (WHO standard)
		80B/20S	80H/20S	80B/20T	80H/20T	25H/55B/20S	25B/55H/20S	25H/55B/20T	25B/55H/20T	
Turbidity (NTU)	13	1.15 ± 0.029	0.8 ± 0.03	0.58 + 0.04	0.5 ± 0.045	1.05 ± 0.03	1 ± 0.03	0.65 ± 0.05	0.45 ± 0.04	5
pH	8.4	8.2 ± 0.10	8.2 ± 0.20	7.9 ± 0.20	7.8 ± 0.20	8.2 ± 0.50	8.4 ± 0.50	7.5 ± 0.80	7.3 ± 0.60	6.5–8.5
Temperature (°C)	25	24 ± 0.00	24 ± 0.00	23 ± 0.10	23 ± 0.10	26 ± 0.00	25 ± 0.00	24 ± 0.20	24 ± 0.10	25
Electrical conductivity (μs.cm^−1^)	1200	1030 ± 33.30	1050 ± 33.20	995 ± 35.00	985 ± 36.20	1030 ± 35.40	1025 ± 36.20	955 ± 35.00	985 ± 36.50	330–1005
Total dissolved solid (mg/l)	1245	530 ± 66.96	515 ± 67.00	415 ± 67.45	400 ± 67.50	480 ± 57.00	465 ± 56.95	360 ± 58.20	395 ± 57.65	200–600
Water hardness (mg/l)	160	105 ± 6.25	94 ± 6.20	85 ± 7.50	82 ± 7.00	102 ± 13.28	96 ± 13.30	75 ± 14.00	78 ± 12.75	60–120
Fluoride Level (mg/l)	3.4	0.085 ± 0.01	0.07 ± 0.00	0.06 ± 0.05	0.065 ± 0.04	0.072 ± 0.00	0.075 ± 0.00	0.055 ± 0.01	0.053 ± 0.01	<1

The turbidity of the groundwater reduced from 13 NTU to 1.15–0.45 NTU, which is ‘below the maximum permissible WHO drinking water standards [21]. The average Total Dissolved Solids (TDS) of filtered groundwater reduced from 1245 mg/l to 360–530 mg/l.

The CPWFs with a composition of 25H/55B/20T and 25B/55H/20T have the highest, and 80B/20S and 80H/20S with the minimum TDS removal efficiencies. All the CPWFs prepared using different compositions have reduced the TDS of the groundwater to the permissible level of the drinking water standard of WHO. The groundwater hardness levels decreased from 160 mg/l to 105-75 mg/l after filtration, i.e., from very hard to moderately hard water levels. The pH and conductivity of filtered groundwater decreased from 8.4 to 7.3 and 1200 μS∙cm^−1^ to 955 μS∙cm^−1^, respectively, which are within the acceptable drinking water standard of WHO [21]. The CPWFs reduced the fluoride level of groundwater from 3.4 mg/l to 0.85–0.053 mg/l. The fluoride removal of CPWFs could be due to the anionic substitution OHˉ with the surface of kaolinite clay (metal oxides) [8–10]. The fluoride level measured after filtration is below the maximum expected range for drinking water (1.5 mg/l) [29].

Moreover, the average Chemical contaminant removal efficiency of the CPWFs was computed and shown in Fig. 5d. The CPWFs show more than an average of 98%, 94%, 60%, and 40% removal efficiency for fluoride level, Turbidity, TDS, and water hardness, respectively.

#### 3.4.3 Microbial removal efficiency

The microbial removal efficiency of the CPWFs fired at 900 °C was tested in the total coliform and fecal coliform using the membrane filtration method, as shown in Table 4.

**Table 4:** The average Total Coliform (TC) and Fecal Coliform (FC) count in colony forming unit before and after filtered groundwater by CPWFs fired at 900 °C.

CPWFs code	Risk Level	Log reduction value (LRV)	Before filtration		After filtration		Removal Efficiency T_C_ (cfu/100 mL) (%)	Removal Efficiency FC (cfu/100 mL) (%)
			TC (cfu/100 mL)	FC (cfu/100 mL)	TC (cfu/100 mL)	FC (cfu/100 mL)		
80B/2OS	Moderate	1.82	840 ± 0.00	132 ± 0.00	8 ± 1.23	2 ± 0.85	99.05	98.48
80H/20S	Moderate	2.12	841 ± 0.00	133 ± 0.00	7 ± 1.20	1 ± 0.76	99.17	99.24
80B/20T	Conformity	3	842 ± 0.00	134 ± 0.00	6 ± 1.45	0 ± 0.36	99.29	100
80H/20T	Conformity	3	843 ± 0.00	135 ± 0.00	5 ± 1.98	0 ± 0.45	99.40	100
25H/55B/20S	Moderate	2.12	844 ± 0.00	136 ± 0.00	7 ± 2.06	1 ± 0.58	99.17	99.24
25B/55H/20S	Moderate	2.12	845 ± 0.00	137 ± 0.00	7 ± 2.00	1 ± 0.58	99.17	99.24
25H/55B/20T	Conformity	3	846 ± 0.00	138 ± 0.00	4 ± 1.50	0 ± 0.95	99.52	100
25B/55H/20T	Conformity	3	847 ± 0.00	139 ± 0.00	3 ± 0.00	0 ± 0.98	99.64	100

Note: TC and FC - stands for total coliform and fecal coliform.

Key: Risk Levels; Conformity <1, Low 1–10,Intermediate 10–100, High 100–1000 and the Categories for microbial removal efficacy are <1 log10 (<90%) is low, 1to 2 log10 (90–99%) is moderate and >2 log10 (>99% is high) (Klarman, 2009).

The number of microbial counts presented in the table is for triplicate Petri dish plates and the computation of the average colony-forming units. The average number of Total Coliform (TC) and Fecal Coliform (FC) counts for the colony-forming unit (CFU) in 0.1 L of raw groundwater was found to be 840 CFU/l and 132 CFU/l, respectively.

The 0.1 L of filtered ground water by CPWF has an average range of 0–8 and 0–2 CFU of the TC and FC, respectively. The total coliform log removal of CPWFs ranges from 1.82 LRV to 3 LRV, which shows the CPWF filer more than 99% of microbes.

Most ceramic water filters are good at filtering larger bacterial organisms but not removing the smaller viral organism. Generally, the prepared CPWFs removed 99.3% of the TC and 98.48% of the FC from the groundwater, and the average microbial counts after filtration fall within the conformity risk level for drinking water quality [21].

## 4 Conclusion

The clay and organic combustible (sawdust and eragrostis tef husk) mixtures and processing parameters optimized for CPWFs production. The percentage of porosity, total shrinkage after firing, and workability of clay and organic additives mixtures are measured. The CPWFs prepared with a weight ratio of 80:20 clay and organic filler show optimum plasticity, mechanical strength, total shrinkage, and porosity. A weight ratio of 80:20 clay and sawdust CPWFs shows a better discharge rate of an average of 36 mL/h.

The Measured physiochemical and microbial removal efficiency results for all CPWF fall within the range of WHO drinking water standards. The CPWFs remove more than 99% of fluoride and microbes from groundwater.

Therefore, this comprehensive research paves the way to manufacture CPWF by small-scale industries using locally available clays that remove microbes and fluoride.

The CPWF will reach, with affordable prices, for the low-income community that drinks untreated groundwater and reduces waterborne health risks.

## 5 Limitation of the study

This study is limited to only analyzing the clay properties, optimizing the processing parameters of CPWF, preparing small-size CPWFs, measuring the discharge rate, and physicochemical and fluoride removal performance due to financial constraint to purchase the necessary large-size molds and related activities. Therefore, we recommend further assessment for the large-size CPWFs, which hold 10 to 20-L water at a time, and have to prepare physiochemical and microbial tests for a longer time, more than months, to satisfy the water need of a family.

## Declarations

### Author contribution statement

Dinsefa Andoshe: Conceived and designed the experiments; Analyzed and interpreted the data; Contributed reagents, materials, analysis tools or data; Wrote the paper. Alemayehu Solomon: Conceived and designed the experiments; Performed the experiments; Analyzed and interpreted the data; Contributed reagents, materials, analysis tools or data; Wrote the paper. Ganesh T, Fekadu Hone, Newaymedhin Tegegne, Tatek Terfasa: Analyzed and interpreted the data. Belay Tesfamariam, Adane Abebe: Contributed reagents, materials, analysis tools or data; Wrote the paper.

### Funding statement

This work was supported by 10.13039/501100015758Adama Science and Technology University (ASTU), Ethiopia.

### Data availability statement

Data included in article/supp. material/referenced in article.

### Declaration of interest's statement

The authors declare no conflict of interest.
